# Supercharged Hypercoagulability: A Case of Heparin-Induced Thrombocytopenia With Thrombosis in a Patient With Double Heterozygous Factor V Leiden and Prothrombin Mutations

**DOI:** 10.1155/crh/3078377

**Published:** 2025-09-28

**Authors:** Yudai Okabe, Jose Ibarra Rodriguez, Jane Edmunds, Nyembezi L. Dhliwayo

**Affiliations:** Division of Hematology and Oncology, Division of Internal Medicine, Rush University Medical Center, Chicago, Illinois, USA

## Abstract

Factor V Leiden (FVL) and the prothrombin 20210A gene mutation are two common genetic predispositions to hypercoagulability. We present a complex case of recurrent venous thromboembolism (VTE) in a 50-year-old woman with double heterozygosity for FVL and prothrombin G20210A, complicated by heparin-induced thrombocytopenia (HIT) and May–Thurner syndrome. Following a recent orthopedic surgery and a sedentary postoperative course, the patient developed extensive bilateral deep vein thrombosis (DVT) and a saddle pulmonary embolism. Initial anticoagulation with heparin was complicated by progressive thrombocytopenia and confirmed HIT, prompting transition to bivalirudin and subsequently argatroban. Despite therapeutic anticoagulation and multiple interventional procedures, the patient experienced repeated thrombotic events. After increasing the therapeutic aPTT goal for argatroban, she ultimately stabilized and was successfully transitioned to oral apixaban. This case highlights the synergistic risk posed by the combination of inherited thrombophilia, structural venous abnormalities, and acquired prothrombotic conditions. It provides insight into the complex nature of proper anticoagulation strategies in these individual cases. Our use of argatroban with higher aPTT goals may provide guidance in future cases of refractory VTE. Further studies are needed to better understand the optimal therapies and management for patients with hereditary and acquired thrombophilia.

## 1. Introduction

Venous thromboembolism (VTE), including deep venous thrombosis (DVT) and pulmonary embolism (PE), is a significant cause of morbidity and mortality. Factor V Leiden (FVL) and the prothrombin G20210A mutation are among the most common inherited thrombophilias genetic predispositions, and heterozygosity for either increases thrombotic risk. Double heterozygosity for both mutations substantially increases this risk, particularly in the presence of additional acquired risk factors including recent surgery, immobility, or infection [1, 2]. We present a patient with double heterozygosity for FVL and prothrombin G20210A, whose course was complicated by recurrent thrombosis, heparin-induced thrombocytopenia (HIT), and May–Thurner syndrome.

## 2. Case Description

We present a case of a 50-year-old Caucasian female with known double heterozygosity for FVL and prothrombin G20210A, who presented with progressive right calf pain and pleuritic chest pain. Two months earlier, the patient had undergone a right acromioclavicular repair, but she did not take her prescribed postoperative prophylactic aspirin 325 mg and was not offered a prophylactic direct oral anticoagulant (DOAC), despite a Caprini VTE score of 14. Her postoperative course was complicated by an upper respiratory infection, contributing to her prolonged immobility and sedentary lifestyle.

**Figure 1:**
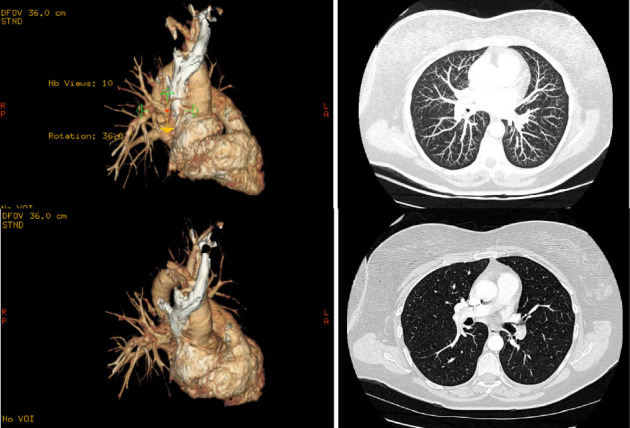
Volume rendering and axial imaging of initial diagnostic CT chest with angiogram protocol showing acute pulmonary sagittal embolus extending into the right and left pulmonary arteries and lobar, segmental, and subsegmental arterial branches bilaterally, with secondary right ventricular heart strain.

In the emergency department, venous duplex revealed acute DVT of the right femoral, popliteal, peroneal, and posterior tibial veins. CT pulmonary angiography demonstrated an acute saddle pulmonary embolus extending into bilateral lobar, segmental, and subsegmental branches without evidence of right heart strain on transthoracic echocardiogram (Figure 1). She was started on intravenous (IV) heparin, and interventional radiology (IR) placed an inferior vena cava (IVC) filter due to high thromboembolic risk. Despite initial intervention and achieving a therapeutic activated partial thromboplastin time (aPTT) goal of 50–75 as per standard hospital protocol, she developed a new acute left common femoral DVT. Repeat duplex on hospital Day 4 demonstrated extensive bilateral thromboses involving the common femoral, deep femoral, superficial femoral, popliteal, posterior tibial, and peroneal veins. Due to the progression of the disease, IR performed bilateral femoral vein, common femoral vein, external iliac vein, and common iliac vein mechanical thrombectomy via AngioJet and balloon angioplasty. Her course was further complicated by progressive thrombocytopenia, with platelets declining from 180 × 10^3^/μL at admission to 38 × 10^3^/μL by day 10. Given the high suspicion for HIT, she was transitioned to bivalirudin. HIT was confirmed with positive platelet factor 4 ELISA (optical density: 1.244) and positive serotonin release assay. Despite initial intervention, she had persistent symptoms and progression of bilateral lower extremity DVTs on serial imaging, prompting transfer to Rush University Medical Center for escalation of care.

On transfer, the patient was transitioned to IV argatroban, hematology and IR were consulted, and serial imaging was obtained. The patient achieved therapeutic aPTT levels (50–75 as per standard hospital protocol) on argatroban. Repeat duplex imaging showed persistent bilateral lower extremity DVTs, and CT angiography of the abdomen and pelvis demonstrated occlusive DVTs extending up to the left common iliac vein with a new partially occlusive thrombus at the IVC filter. The patient subsequently underwent a venogram that revealed extensive thrombosis of the bilateral lower extremities and May–Thurner compression of the left common iliac vein. Due to the persistent thrombosis, IR performed a mechanical, pharmacomechanical, and suction thrombectomy using a ClotRetriever, FlowTriever, and adjunctive tissue plasminogen activator with balloon maceration. During this procedure, the thrombosed IVC filter was removed; stents were also placed in the left common and external iliac veins. Dual antiplatelet therapy was initiated with aspirin 81 mg daily and clopidogrel (300 mg loading dose, then 75 mg daily). This procedure was complicated by an IVC hematoma and subsequent rethrombosis in her bilateral lower extremity veins depsite therapeutic anticoagulatin after the procedure.

Due to continued clot burden, venogram was repeated 7 days later. This demonstrated occlusive thrombus from the bilateral posterior tibial to popliteal veins and residual thrombus within the left iliac stent. IR performed suction thrombectomy of the left iliac stent and popliteal vein, balloon venoplasty of the left popliteal vein and iliac stent, and bilateral thrombolysis. Postlysis venogram revealed patent femoral, popliteal, and iliac veins bilaterally with brisk antegrade IVC flow, minimal nonflow-limiting thrombus in the left iliac stent, and only residual infrapopliteal thrombus. She was transitioned to oral dabigatran 150 mg twice daily, given the improved symptoms and venous flow. However, 4 days later, she developed recurrent left lower extremity pain, and a repeat duplex ultrasound revealed extensive, nearly occlusive thrombus extending from the left distal external iliac through the calf veins. The right lower extremity clot burden remained stable. She was transitioned back to IV argatroban. Because of persistent thrombosis despite previously achieving standard therapeutic aPTT levels, the aPTT goal was increased to 80–90 to further optimize anticoagulation. The patient underwent IR-guided venogram, AngioJet pulse spray, and venoplasty of the left external iliac, common iliac vein stent. Subsequent left lower extremity duplex imaging showed a stable clot burden, and the patient was transitioned to oral apixaban with no further thrombotic events.

The patient ultimately stabilized on IV argatroban after setting the higher aPTT goal (80–90). Of note, platelet counts improved during the rest of the hospital following transition to nonheparin products, rising to 80 × 10^3^/μL by Day 3, 165 × 10^3^/μL by Day 6, and 392 × 10^3^/μL by Day 7 after transition. The patient was successfully transitioned to apixaban as per current guidelines with no further concerns for recurrent VTE and recurrent symptoms. She was discharged from the hospital with apixaban 5 mg twice daily along with dual antiplatelet therapy (aspirin 81 mg daily and clopidogrel 75 mg daily).

## 3. Discussion

### 3.1. Current Understanding of Our Case

This case highlights the complexity of managing patients with double heterozygosity for FVL and prothrombin G20210A when additional risk factors for thromboembolism are present. Her initial clinical picture is a common presentation of VTE. It is known that patients with thrombophilia have an increased risk for VTE following joint surgery [3]^.^ This risk was further amplified by postoperative immobility, inflammation from a concurrent upper respiratory infection, and nonadherence to prescribed aspirin prophylaxis.

Management was further complicated by the diagnosis of HIT and May–Thurner syndrome, which further contributed to recurrent thrombosis despite adequate anticoagulation and multiple interventions. Our initial understanding of this case was that her initial treatments failed due to the onset of HIT. This hypothesis is further supported by the successful thrombectomy and thrombolysis that were performed after her platelets had fully recovered.

This initial theory was complicated when an attempt to transition her from IV argatroban to oral dabigatran failed due to rethrombosis in her left lower extremity. Our team briefly considered the use of IVIg for treatment of refractory HIT, which has been previously described in a small case series and review that showed clinical resolution of HIT with IVIg (1 g/kg) [4, 5]. However, our clinical suspicion for refractory HIT remained low given that her platelet counts remained stable throughout. Likewise, we had low concern for dabigatran failure, as the right lower extremity thrombus burden remained unchanged. Instead, we suspect the progression of her thrombosis was multifactorial, including anatomical abnormalities from May–Thurner syndrome and iatrogenic factors related to recurrent interventional procedures.

Notably, this case reflects the interplay between genetic predisposition and structural anomalies in thrombotic risk. As described by Buxbaum et al., coexisting thrombophilia and May–Thurner syndrome can significantly increase the risk of extensive and recurrent thrombotic events, which may explain the complexity seen in our patient [6]. Similarly, Alhassani et al. discuss the challenges of managing such patients and emphasize a personalized approach due to variability in anticoagulation response and clinical outcomes [7].

### 3.2. Similar Cases

Unfortunately, no prior case reports have documented individuals who are double heterozygous for FVL and prothrombin G20210A with HIT and May–Thurner syndrome that we could compare our patient's clinical course to. No comprehensive study has examined the coexistence of all three conditions. However, multiple studies have characterized important components of this patient's condition.

The increased risk for VTE in individuals with double heterozygosity for FVL and prothrombin G20210A is well established. Ryu et al. identified 662 individuals with double heterozygous mutations and found that these individuals had a significantly increased risk for VTE compared to those without the mutation, with an odds ratio of 5.24 (95% CI: 4.01–6.84) [8]. A retrospective cohort study of 138 female double heterozygotes similarly demonstrated a higher VTE risk compared to single heterozygotes and wild-type carriers, with hazard ratios of 2.51 (95% CI: 1.55–4.08) and 2.53 (95% CI: 1.58–4.05), respectively [9]. Another study of 100 individuals with compound FVL and prothrombin G20210A found that 50% of individuals experienced their first VTE by age 41, with 38.2% unprovoked [10]. De Stefano et al. evaluated 412 patients, who were referred after their first VTE, and found that individuals who were heterozygous for both FVL and prothrombin G20210A were at much higher risk for recurrent thrombotic events. Specifically, the relative risk of recurrent thrombotic events in individuals who were heterozygous for both FVL and prothrombin G20210A compared to FVL alone and wild-type individuals was 2.6 (95% CI: 1.3–5.1) and 2.7 (95% CI: 1.4–5.0), respectively [2].

No study has investigated the specific relationship between May–Thurner syndrome and double heterozygous patients. However, it is known that May–Thurner syndrome independently predisposes patients to thrombosis due to the compression of the left common iliac vein by the right common iliac artery. A recent imaging-based study also suggests that venous geometry serves as an independent risk factor for VTE. A study titled *“Correlation between common iliac vein geometry and the risk of deep vein thrombosis in patients with May–Thurner syndrome”* found that geometric variations in the iliac vein, such as greater angulation, cross-sectional narrowing, and reduced flow area, correlated significantly with thrombotic risk even when controlling for the degree of compression [11]. Vascular architecture is an important factor in assessing thrombotic risk and likely contributed to our patient's recurrent VTE.

Data on the coexistence of inherited thrombophilia and HIT are limited. There is one retrospective study that evaluated 165 individuals with serologically confirmed HIT for the presence of FVL and demonstrated that individuals with FVL were not at increased risk for HIT [12]. However, isolated case reports highlight similar scenarios to our patient. John et al. described a 17-year-old with congenital renal disease and double heterozygosity for FVL and prothrombin G20210A who developed HIT after chronic heparin exposure during dialysis. The patient was started on heparin during their dialysis sessions for prophylactic anticoagulation. The patient subsequently developed HIT 8 months after heparin was first initiated. The care team transitioned the patient's anticoagulation to lepirudin, which resolved the thrombocytopenia, prevented future thrombotic events, and ultimately allowed the patient to receive dialysis without further complications [13]. This case provides insight into the successful use of lepirudin in controlling future thromboembolic events in a patient similar to our case.

Determining an anticoagulation regimen for our patient was challenging due to the concurrent HIT and multiple recurrent thrombotic events. Our patient experienced thrombus progression on bivalirudin after initial HIT diagnosis and continued to experience progression of disease despite transitioning to argatroban. The American Society of Hematology 2018 guidelines recommend the use of argatroban, danaparoid, fondaparinux, and bivalirudin in cases of HIT, with a previous meta-analysis including 4698 cases showing no significant differences in safety and efficacy across agents, including DOACs [14, 15].

This study also showed that DOACs may provide a favorable outcome, including platelet recovery in 96%, recurrent thromboembolism in 3%, and major bleeding in 1% of HIT cases, though there was no statistical significance. Regarding anticoagulation in patients who are double heterozygous such as our patient, the American College of Physicians recommends the use of DOACs for the prevention of thromboembolism. Zuk et al. specifically analyzed individual DOACs in a single-center study with 56 cases of VTE in patients with inherited thrombophilia. They found that rivaroxaban was associated with increased risk of recurrent VTE and bleeding compared to apixaban or dabigatran (hazard ratio: 2.76; 95% CI: 1.26–3.92) [16].

## 4. Conclusion

No single study or guideline adequately describes the best regimen for the rare coexistence of double heterozygosity for FVL and prothrombin G20210A, May–Thurner syndrome, and HIT as seen in our patient. In this case, previous guidelines and prior studies were utilized to guide the clinical decision-making process. As demonstrated by previous studies and case reports, our patient had multiple factors that significantly increased the risks for VTE, which all contributed to her complicated course. In addition, the thrombotic events were difficult to control on previously established therapeutic regimens and guidelines. Ultimately, the use of IV argatroban with stricter and higher aPTT goals stabilized the patient. This case highlights the potential role of escalating therapeutic goals for anticoagulation in similar cases,, providing guidance for clinicians faced with similar challenging cases. Further studies are necessary to better understand the safety and efficacy of higher aPTT goals as well as the optimal therapies in similar, refractory cases of VTE in patients with multiple inherited and acquired prothrombotic risk factors.
